# Anticipation and Realisation

**Published:** 1920-12-18

**Authors:** 


### THE COLLEGE OF NURSING.

### Anticipation and Realisation.

THE chief disadvantage from which the College has suffered since its foundation is absence of criticism. Of abuse there was abundance, but of constructive criticism there has been, for all practical purposes, none. The main purpose that abuse serves is to gratify its author, whereas criticism is the first duty of truest friendship. Very old and very authoritative is the warning "woe unto you when all men shall speak well of you." As Lord Fisher remarks, "when criticism goes, life is done! You must squeeze the fragrant leaf to get the delicious scent."

Speaking for myself, I have ever been a strong supporter of the College. It came into being at a time when the nursing profession was rent with faction and bitter dissension, and when, at the same time, British nurses were wholeheartedly offering their indispensable services to the Red Cross, military and civil. My conviction has never been shaken that the founders of the College were inspired by the highest motives, and that their determination was to do all within their power to help the profession. The programme was vague and undefined as regards time or method of procedure, but it included economic, educational, and social reform. There was, indeed, in contemplation something wider and more alluring even than this. British nurses overseas were to be gathered into a union which would tend to promote comradeship and prosperity amongst all those who wore the mantle of Florence Nightingale within the King's realms. Nor was there any serious obstacle in the way to complete achievement. As I pointed out again and again in this connection, the nation thought as highly of its nurses as it did of its sailors, soldiers, and flying men. They won their honours on the field, and the College had nothing to do but see that these were duly conferred. The opportunity was unique. It was magnificent. So was the machinery. But one thing needful was missing. The Executive lacked energy, initiative, enterprise, vitality.

I have long outlived the age of saying one thing and meaning another. Nothing is to be gained by using euphemisms. The ladies who were put on the Council were, without exception, splendid as matrons, but for the purpose outlined they were utterly unfit.

Moreover, I do not believe that any serious effort to achieve was ever made. There is no evidence that would lead to any other conclusion. I believe that what a large number of the matrons deliberately set out to do, inspired with the highest motives, was to allay agitation amongst nurses by promising them wonderful achievements at a later and altogether undefined date. Tibbs eve, in fact. When all the world was reconstructing the Nurse was doped with shibboleths about sacrifice, vocation, devotion, and what not.

A matron, able to organise a hospital staff and to maintain the highest efficiency in its working, is not necessarily an agitator. She is probably the very reverse. I am using plain words, and I have no hesitation in affirming that none but an agitator is of use on the Council of the College. No great public reform was ever achieved without agitation. The trumpet has to be sounded, and all the people have to shout together. Otherwise the walls of Jericho will stand fast forever.

What did I expect? To be precise, in the first place I expected hum. It takes a staff of at least half-a-dozen working all day and at full steam to run a small shop in the suburbs. If you doubt it, ask the first greengrocer you meet, and when you have satisfied yourself, work out a little sum in Rule of Three. How many full-time workers are necessary to run an institution with twenty thousand members, scattered all over the kingdom, with a potentiality of fifty or even a hundred thousand? There are small exclusive clubs in the neighbourhood of the College employing far more and better paid full-time workers than the College employs, and none of them has a sinecure job. I expected hum, but I can record only hum-drum. The matrons of the Council are busy women, hard at work all day and every day, and even if they had the wish they have not the strength to accomplish what the College owes to the women workers with whose subscriptions it was built.

### READ FISHER'S RECORDS.

There is a remarkable similarity between the mentality of the old Naval admirals as described by Lord Fisher and the College matrons. I wish they would read Lord Fisher's Records. They will find themselves on every page. Here are one or two extracts: "I heard one First Sea Lord say to the Second Sea Lord, when scandalised at seeing in a new ship a bathroom for the midshipmen, that he never washed when he went to sea, and didn't see why the midshipmen should now."

"What my retrograde enemies perfectly detested was being called the 'bow and arrow' party. When later they fought me about speed being the first desideratum, the only way I bowled them over was by designating them 'the Snail and Tortoise party.'"

One of the most telling stands alone and without comment:

"Ye men of Galilee! Why stand ye gazing up into Heaven?"

IERNE.

